# The latent structure of ICD-11 Prolonged Grief: Replicated factor mixture models in two national cohorts

**DOI:** 10.1371/journal.pmen.0000515

**Published:** 2026-02-20

**Authors:** James Cunningham, Mark Shevlin, Eoin McElroy

**Affiliations:** School of Psychology, Ulster University, Coleraine, United Kingdom; National University of Singapore, SINGAPORE

## Abstract

Prolonged Grief Disorder (PGD) is recognised in ICD-11 and DSM-5-TR, yet its latent structure in the general population remains uncertain. We examined whether population-level prolonged grief, assessed with the International Prolonged Grief Disorder Scale (IPGDS), is best characterised by a single severity continuum or a hybrid structure combining dimensions with latent classes, using cross-sectional data from two independent general-population cohorts: a UK community panel (N = 1,777) and a nationally recruited Irish sample (N = 950). Analyses followed a stepwise sequence of exploratory and confirmatory factor analyses, latent profile analysis, and factor mixture modelling. In both cohorts, a three-factor measurement structure was supported, with Items 5–8 (anger, avoidance, blame, difficulty accepting) showing consistent cross-sample instability. A three-factor, four-class factor-mixture model provided the most interpretable and reproducible summary across samples, preserving the factor structure while allowing latent classes to accommodate atypical item behaviour. The same four severity-ordered classes and prevalence ranking were recovered in both cohorts, with high classification precision (entropy = 1.00; average posterior probabilities ≥.97). Symptom profiles progressed monotonically with severity; blame and anger functioned as high-severity indicators, whereas avoidance contributed little to distinguishing the highest-severity class. At the population level, PGD symptoms were primarily dimensional, with latent classes best interpreted as pragmatic severity groupings rather than discrete categories. For surveillance and screening, dimensional assessment focused on separation distress may be complemented by brief checks for persistent blame and anger, with avoidance of lower discriminative value in community samples. As these findings are based on cross-sectional data, future longitudinal research is needed to test the temporal stability of class membership and its prognostic value for functional outcomes and service use.

## Introduction

Bereavement is universal, yet a sizeable minority experience persistent yearning, preoccupation, emotional numbness, difficulty accepting the loss, and functional interference that exceed culturally expected mourning periods and impair daily life [1–3]. Prolonged Grief Disorder (PGD) is now recognized in ICD-11 and DSM-5-TR, providing a shared nosological frame that has accelerated measurement, validation, and population surveillance [4,5]. In both systems the diagnosis emphasizes enduring symptoms with associated distress and impairment, which has helped standardize inclusion criteria and prompted more consistent questionnaire development and translation. Despite this harmonization, a central question for population research remains unresolved: does PGD represent a qualitatively distinct condition, or primarily the high end of a general grief severity continuum [1,6–8]?.

Across psychopathology, dimensional models often better capture graded symptom burden and comorbidity, reduce reliance on arbitrary cut-points, and improve population-level inference and surveillance [9–11]. Dimensional scores also align well with screening, where sensitivity and specificity can be tuned to the task, whereas categorical and hybrid approaches retain value when they identify subgroups that differ not only in severity but also in symptom configuration, prognosis, or likely intervention needs [12,13]. In applied settings, such subgroups can sharpen clinical communication and inform stepped care when profiles carry distinct patterns of impairment or treatment indication. For PGD, the key empirical task is to determine whether heterogeneity in community samples reflects smooth severity differences or discrete subpopulations with distinct symptom profiles. This distinction matters for communication, screening thresholds, and triage.

Taxometric and dimensional-spectrum research provides important context for this debate. Taxometric studies of prolonged grief symptoms in community samples have consistently failed to identify evidence for a discrete grief taxon, instead supporting a continuously distributed severity construct (14,7). More broadly, dimensional models of psychopathology emphasize graded symptom severity and shared liability across disorders, cautioning against the imposition of categorical boundaries when empirical support is weak (9, 15). At the same time, theoretical perspectives such as cognitive–attachment models of grief suggest that qualitative differences in meaning-making, attachment representations, and coping processes may emerge at higher levels of symptom severity (16, 13). Factor mixture models are uniquely suited to adjudicate between these possibilities by allowing simultaneous estimation of continuous latent dimensions and discrete latent classes, thereby providing a principled test of whether observed heterogeneity reflects severity bands alone or the presence of qualitatively distinct subgroups.

The evolution of post traumatic stress disorder (PTSD) illustrates how such constructs can move from unidimensional to multidimensional and hybrid structures. PTSD initially appeared in DSM- III as a unitary category anchored by intrusive memories, avoidance, and arousal [14]. Factor analytic work helped refined its structure, distinguishing intrusion, avoidance, negative alterations in cognition and mood, and arousal, thereby improving measurement and interpretability [15,16]. Person-centered analyses also identified interpretable subgroups, including a dissociative profile recovered via latent class or profile analysis that differs meaningfully in presentation and risk [17]. Confirmatory models further supported richer multidimensional structures, for example a seven factor solution incorporating anhedonia and externalizing behaviors that captured additional variation in symptom clustering [18]. Expanded factor solutions have been critiqued for limited clinical utility and potential overfitting [19,20]. Hybrid models still add value by showing how severity gradients and subtypes can coexist. Using factor mixture modeling, large sample work has recovered ICD 11 consistent PTSD and Complex PTSD structures while identifying distinct classes in population data [21]. These developments motivate parallel tests in PGD, where similar tensions between severity and subtype likely arise.

Parallels between PTSD and PGD are clear. Both follow major life events, involve intrusive cognitions and avoidance, and show substantial heterogeneity across individuals, relationships, and cultures [3,17,22]. For PGD specifically, latent variable investigations report compact severity factors and separable symptom clusters, with evidence of cross-cultural variability; however, findings vary by instrument, sample, and modeling choices [23,24]. This inconsistency complicates public health messaging and screening, where simple, robust rules are needed to balance overidentification against missed need. A further limitation of this literature is that grief-related contexts may be insufficiently captured by standardised symptom measures, particularly in non-Western or culturally diverse populations. Symptom endorsement can plausibly be shaped by culturally normative beliefs about responsibility, emotional restraint, continuing bonds, and social role obligations, rather than severity alone. Items assessing constructs such as blame, avoidance, or difficulty accepting the loss may therefore function differently depending on cultural norms around meaning-making, moral responsibility, and acceptable expressions of distress. If such contextual influences are not explicitly modelled, latent structures may appear artificially unidimensional or unstable, potentially obscuring meaningful heterogeneity in how grief is experienced and expressed across populations. This underscores the importance of testing measurement models across diverse contexts rather than assuming cross-cultural equivalence by default. If severity is primarily graded, cut points should be purpose specific and transparent about trade offs. If distinct classes exist, communication and supports may need to reflect profile specific patterns relevant to burden and functioning.

Measurement choices are central. The International Prolonged Grief Disorder Scale (IPGDS) (Killikelly et al., 2020) was designed to align directly with ICD 11, capturing longing and preoccupation alongside accessory symptoms and functional impairment, with culture adaptation items to support cross national work [25]. Initial studies report acceptable reliability and convergent validity, including in non-English-speaking contexts [25,26]. However, many analyses rely on modest samples or simple factor or profile models that cannot adjudicate between purely dimensional and hybrid structures. Recent syntheses emphasize the need for stronger modeling of latent structure, clearer reporting of class distinction and assignment quality, and improved comparability of symptom profiles across studies to enable population level conclusions [27,28]. Methodological work further recommends simulation informed class enumeration procedures and transparent reporting of separation and assignment indices when mixture solutions are proposed, so that any claimed subtypes can be evaluated for practical distinctiveness and reproducibility [29].

Accordingly, we directly tested these issues in two independent general-population cohorts from the United Kingdom and Ireland, evaluating whether ICD-11 PGD symptoms are best captured by a single severity continuum or a hybrid structure with latent classes. Sample 1 was the C19PRC-UK Wave 5 panel. Sample 2 was a nationally recruited Irish Qualtrics cohort. This dual-cohort design enabled direct replication of the selected model and evaluation of stability across national contexts, a step frequently recommended but rarely implemented in latent-variable grief research. We examined three targets of inference. First, cross-cohort reproducibility of symptom structure using exploratory and confirmatory factor models. Second, the distinctiveness and assignment precision of recovered classes, indexed by entropy and class-specific posterior probability accuracy. Third, epidemiologically interpretable class sizes and within-class symptom means and variances. By prioritizing replication, assignment quality, and profile interpretability, we seek to minimize the risk of spurious or overfitted class solutions that complicate the existing literature.

The overarching objective was not to establish new diagnostic thresholds for clinical practice, but to deliver an epidemiologically useful account of prolonged grief symptom organization that informs population surveillance, public health communication, and the refinement of screening tools [30,31]. In doing so, the study directly addresses calls for advanced mixture modeling in PGD, extends prior work on dimensional versus categorical organization of grief, and provides one of the first systematic cross national tests of ICD-11 PGD symptom structure using factor mixture models.

## Materials and methods

### Ethics statement

All procedures were reviewed and approved by the relevant institutional research ethics committees. The C19PRC-UK study was approved by the University of Sheffield Research Ethics Committee, and the Irish cohort protocol was approved by Ulster University’s Research Ethics Committee (RG3-FCPSY-22–026-A).

All participants were aged 18 years or older and provided informed consent online before taking part. Both datasets were fully anonymised before being accessed by the authors, and no member of the research team had access to any direct or indirect identifiers (including names, email addresses, IP addresses or contact details).

The anonymised UK dataset was accessed for research purposes on 14 July 2025, and the anonymised Ireland dataset was accessed on 25 July 2025. The study complied with the Declaration of Helsinki, the EU General Data Protection Regulation (GDPR) and all applicable data-protection requirements while following the STROBE guidelines for observational studies (S1 STROBE Checklist). De-identified data underlying the findings reported in this study are publicly available via the following link: https://osf.io/e8xst/overview?view_only=896b124d298848cfa5ad0b26331e7e4a

## Participants

### Sample 1: C19PRC-UK Wave 5 (United Kingdom)

The first sample came from Wave 5 of the UK COVID-19 Psychological Research Consortium (C19PRC-UK), a nationally representative panel of adults aged 18 years and older living in the UK [32]. Data were collected in March to April 2021 using quota sampling on sex, age and household income. Of 2,520 respondents, 1,944 reported the death of someone close at any point in life. Participants bereaved within the past < 6 months were excluded (n = 167), and a further 14 were excluded due to missing key variables, yielding a final analytic sample of N = 1,777. Ethical approval was granted by the University of Sheffield Research Ethics Committee. The research team accessed the fully anonymised dataset on 14 July 2025, and no identifiable information was available at any time. Sample characteristics are summarised in Table 1 (mean age = 54.53, SD = 14.26; IPGDS M = 22.14, SD = 10.04).

**Table 1:** Sociodemographic (Weighted) Characteristics of the Sample (N = 1777).

	% (n)
**Sex**	
Male	50.0 (888)
Female	49.7 (883)
Transgender/ Prefer not to say	0.3 (6)
**Age in years**	
18–24	2.0 (36)
25–34	9.6 (170)
35–44	13.9 (247)
45–54	19.6 (349)
55–64	27.2 (483)
65+	27.7 (492)
Age	M = 54.53, SD = 14.26
**Region**	
England	52.2 (928)
Wales	17.1 (303)
Scotland	18.8 (334)
Northern Ireland	11.9 (212)
**Current relationship status**	
In a committed relationship	62.9 (1119)
Not in a committed relationship	37.1 (658)
**Current employment status**	
Employed	49.6 (882)
Unemployed	18.6 (331)
Retired	31.7 (564)
**IPGDS Total Score**	M = 22.14, SD = 10.04

### Sample 2: Qualtrics panel (Republic of Ireland)

The second sample comprised bereaved adults recruited in the Republic of Ireland via Qualtrics double opt-in research panels. Eligibility required age ≥ 18 years and endorsement of a past bereavement. Data were collected from 21 April to 12 September 2022. After applying the ≥ 6-months-since-bereavement criterion and excluding participants with missing key variables, the final analytic sample was N = 950. Ethical approval was provided by Ulster University’s Research Ethics Committee (RG3-FCPSY-22–026-A). The research team accessed the fully anonymised dataset on 25 July 2025, and no author had access to any information that could identify participants during or after data collection. Sample characteristics are shown in Table 2 (mean age = 45.36, SD = 15.39; IPGDS M = 15.62, SD = 10.68).

**Table 2:** Sociodemographic (Weighted) Characteristics of the Sample (N = 950).

	% (n)
**Sex**	
Male	47.2 (448)
Female	52.7 (501)
**Age in years**	
18–24	8.5 (81)
25–34	20.1 (191)
35–44	19.8 (188)
45–54	19.6 (186)
55+	32.0 (304)
Age	M = 45.36, SD = 15.39
**Current employment status**	
Employed	68.2 (649)
Unemployed	11.1 (105)
Retired	13.2 (125)
Student	4.2 (40)
Disabled	3.3 (31)
**IPGDS Total Score**	M = 15.62, SD = 10.68

### Measures

The International Prolonged Grief Disorder Scale (IPGDS) is a self-report instrument designed to assess ICD-11 PGD symptoms [28]. The scale consists of 14 items: two core symptoms (longing, preoccupation), ten emotional-distress items, one item assessing functional impairment, and one item evaluating whether symptoms exceed sociocultural norms. Responses are given on a five-point Likert scale from 1 (“Not at all”) to 5 (“Always”). In the Irish sample (N = 950), items were originally coded 0–4; to ensure comparability with the UK sample (1–5), responses were rescaled to a 1–5 metric prior to analysis by adding one point to each item score. For latent-structure analyses we modelled the 12 symptom items (excluding impairment and the cultural criterion) to avoid mixing consequence and cultural appraisal with symptom content. Internal consistency across the 12 items was excellent (α =.94) [28]. Total symptom scores ranged 12–60, with higher scores reflecting greater PGD severity.

### Statistical analysis

Descriptive statistics were run in SPSS 27 to check the 12-item IPGDS and to examine bivariate links with demographic and loss-related variables. After data screening, N = 1,777 cases were available in Sample 1. A single missing-data pattern and full covariance coverage (1.00) supported full information maximum likelihood (FIML) with a robust MLR estimator in Mplus 8.1 [33]. Item distributions showed moderate skew (<= 2.1) and kurtosis (<= 3.8). No univariate or multivariate outliers exceeded common cut-offs, so no transformations or exclusions were needed.

To study the latent structure of ICD-11 PGD symptoms, we used a stepwise plan. Exploratory factor analysis (EFA) tested one- to four-factor solutions for the 12 items with geomin rotation (ε = 0.01) and allowed factors to correlate. We kept a factor when each factor had at least two salient loadings (≥.40), and the pattern made conceptual sense. Salient loadings were defined a priori as standardized loadings ≥ .40 on a single factor, with cross-loadings considered problematic when an item loaded ≥ .30 on more than one factor or failed to show a clear primary loading. Factor solutions were evaluated not only on global fit indices but also on simple structure, conceptual coherence, and interpretability, consistent with recommended best practice in exploratory factor analysis. Local instability was identified when items showed weak primary loadings, diffuse cross-loadings, or inconsistent loading patterns across samples. Given the correlated nature of grief symptoms, geomin rotation was selected to allow factors to correlate while penalising complex loading patterns; alternative orthogonal rotations were not pursued as they impose unrealistic independence assumptions for bereavement-related constructs. Four-factor EFA solutions were rejected when additional factors failed to meet retention criteria, specifically when a factor was defined by fewer than two salient loadings or relied primarily on cross-loading items, despite marginal improvements in global fit.

Confirmatory factor analysis (CFA) then compared a one-factor model, the ICD-11 core-versus-emotional two-factor model, and alternative three-factor models from recent work [23,24]. The model evaluation focused on replicated loading patterns and local misfit concentrated in Items 5–8; modification indices were reviewed descriptively to characterise sources of misfit, but model retention prioritised cross cohort replication and interpretability over adding sample specific correlated residuals.A four-factor option found in our EFA was also tested.

Latent profile analysis (LPA) looked for homogeneous response groups by estimating two- through six-class models (STARTS = 7500 1500; ALGORITHM = INTEGRATION). We kept models when profiles were interpretable, the Lo-Mendell-Rubin adjusted likelihood ratio test (LMR-A) was significant versus k − 1 classes [34], each class was at least 5 percent of the sample, and classification quality was adequate based on entropy and class-specific average posterior probabilities [30]. Given that very high entropy values can reflect over-separation or local solutions in mixture models, we explicitly examined classification diagnostics beyond entropy alone. This included inspection of class-specific average posterior probabilities, evaluation of multiple random start solutions to reduce the risk of local maxima, and checks for solution stability across estimation runs.

Factor mixture models (FMM) then combined the best factor measurement structure with class estimation, using the same random-start settings, to test whether PGD is best seen as a single severity continuum or as distinct subtypes [12].

Model evaluation used several checks. For EFA and CFA, and where relevant for LPA and FMM, we recorded the Comparative Fit Index (CFI) [34], Tucker-Lewis Index (TLI) [35], Root Mean Square Error of Approximation (RMSEA) [36], and Standardised Root Mean Square Residual (SRMR) [37]. We noted common descriptive cut-offs (for example, CFI/TLI >= 0.90 acceptable and>= 0.95 excellent; RMSEA <= 0.08 acceptable and <= 0.05 close fit) [38]. We also compared AIC, BIC, and sample-size-adjusted BIC for simpler, better-fitting models [39,40]. For mixtures, we examined entropy, class-specific average posterior probabilities, and TECH11/TECH14 outputs; a non-significant Vuong or adjusted LMR test meant no gain from adding another class.

Decision rules reflected our public health focus. We prioritised: (i) replication of the three-factor measurement structure across cohorts; (ii) clear class separation and precise assignment (entropy and posterior-probability accuracy); (iii) population interpretability, including stable class prevalences and within-class symptom means and variances; and (iv) statistical support from LMR-A with backing from information criteria. Fit indices and information criteria are reported in S1 Table and S2 Table for EFA, CFA, LPA, and FMM in Sample 1, and for the Irish cohort where relevant. The narrative focuses on replication, separation, and interpretability. When competing solutions yielded small improvements in global fit, preference was given to the most parsimonious structure that replicated across cohorts and satisfied simple structure criteria.

Two robustness checks increased confidence in the chosen solution. First, we re-estimated the best CFA with WLSMV on polychoric correlations and found near-identical loadings and fit. Second, allowing class-specific factor variances in FMM did not improve simplicity or stability, so variances were held equal. Overall, model choice was based on converging evidence from fit indices, replication, simplicity, and population interpretability, consistent with prior latent-variable work on ICD-11 trauma-related constructs [21,41,42] and recent guidance for PGD research [23].

## Results

### Measurement models: EFA

In both samples, the one-factor model fit poorly, and overall fit improved as additional factors were added. UK (N = 1,777): 1-factor CFI = .854, TLI = .822, RMSEA = .116; 2-factor CFI = .958, TLI = .936, RMSEA = .069; 3-factor CFI = .989, TLI = .978, RMSEA = .040; a 4-factor model further improved indices (CFI = .998, TLI = .995, RMSEA = .020) but was not retained because the extra factor was poorly defined (one clear loading plus a cross-loading). Although global fit indices improved with four factors, the additional factor did not satisfy prespecified retention criteria and undermined interpretability, indicating statistical overextraction rather than substantively meaningful structure.

Ireland (N = 950): 1-factor CFI = .840, RMSEA = .128; 2-factor CFI = .964, TLI = .945, RMSEA = .068; 3-factor CFI = .991, TLI = .982, RMSEA = .039; the 4-factor model improved global indices but showed the same interpretability problem, so the 3-factor solution was retained. In both datasets, a local instability centred on Items 5–8 (anger, avoidance, blame, difficulty accepting), which showed weaker or cross-loadings; items outside this band loaded coherently. Full fit statistics are provided in S1 Table and S2 Table. Prior to model testing, descriptive characteristics of the IPGDS in both cohorts are shown in Table 3**.**

**Table 3:** Descriptive Statistics for International Prolonged Grief Disorder Scale (IPGDS).

Item	*M*	*SD*	*Endorsement*	*M*	*SD*	*Endorsement*
	***UK***			***Ireland***		
1: Yearning	2.33	1.13	12.9	2.94	1.11	26.8
2: Preoccupation	2.00	1.06	8.4	2.52	1.15	19.1
3: Feelings of Sorrow	2.28	1.19	15.5	2.91	1.19	30.0
4: Feeling Guilty	1.72	1.05	7.7	2.10	1.25	15.0
5: Anger	1.90	1.17	10.7	2.45	1.31	21.9
6: Avoid Reminders	1.70	1.04	7.1	2.09	1.21	14.7
7: Blame Others	1.43	0.90	4.7	1.65	1.07	8.5
8: Trouble Accepting Loss	1.73	1.08	7.9	2.29	1.26	16.7
9: Loss of Self	2.23	1.30	16.5	2.80	1.33	28.9
10: No Desire for Joy	1.63	1.02	7.1	1.87	1.20	10.6
11: Emotional Numbness	1.70	1.03	6.7	2.08	1.24	15.0
12: Activity Difficulties	1.54	0.93	5.0	1.93	1.17	12.8
13: Functional Impairment	1.47	0.89	4.5	1.79	1.02	8.6
14: Worse than Others	1.48	0.93	5.5	1.58	0.82	0.0

Note. M = mean; SD = standard deviation; Endorsement = percentage endorsing the item at 4 (Often) or 5 (Always) (i.e., responses > 3 on the 1–5 scale). This threshold was used to summarise higher frequency symptom endorsement consistent with common practice for Likert type symptom items and to avoid inflating endorsement rates by including the midpoint (3, Sometimes).

### Measurement models: CFA

Confirmatory models in both samples favoured the three-factor specification over one- or two-factor alternatives, with stable loadings outside Items 5–8 (anger, avoidance, blame, difficulty accepting); Item 7 (blame) was least stable.

UK (N = 1,777): 1-factor CFI = .857, TLI = .822, RMSEA = .115, SRMR = .056; 2-factor CFI = .920, TLI = .896, RMSEA = .088, SRMR = .054; 3 factor CFA (CFI = .944, TLI = .932, RMSEA = .071, SRMR = .164), with information criteria of AIC = 48285.800 and BIC = 48483.177).

Republic of Ireland (N = 950): 1-factor CFI = .840, TLI = .804, RMSEA = .128, SRMR = .062; 2-factor CFI = .904, TLI = .885, RMSEA = .098, SRMR = .125; 3-factor CFI = .944, TLI = .931, RMSEA = .076, SRMR = .122, with lower AIC (29825.26) and BIC (30000.09) values than the one- and two-factor models. Full CFA fit tables are provided in S1 Table and S2 Table. Standardized CFA factor loadings for the retained three-factor IPGDS model in each cohort are reported in S3 Table, with items mapped to Separation distress, Emotional reactivity, and Emotional numbness.

### LPA results

Latent profile analysis (LPA) was used to identify subgroups based on PGD symptom profiles. In both samples, solutions from two to six classes were tested, and the four-class model provided the clearest balance of fit, separation, and interpretability.

UK (N = 1,777): the LMR-A was significant up to four classes (p < .001) but not for the five-class model (p = .1565). Although the six-class model was significant (p < .001), the improvement was marginal and class separation weakened. The four-class solution showed good fit and clear structure, with AIC = 48191.85, BIC = 48537.26, and entropy = .930, indicating strong classification precision. Information criteria continued to decline slightly beyond four classes, but interpretability worsened, so the four-class model was retained.

Republic of Ireland (N = 950): the LMR-A supported the two-class (p < .001) and four-class (p = .017) models but not the three-, five-, or six-class solutions (p values = .063–.293). Entropy ranged from.878 to.943 across models, declining slightly as classes were added. The four-class model again achieved the best balance of fit and clarity, with AIC = 30041.12, BIC = 30347.07, and entropy = .900. Although AIC/BIC values fell further in larger models, improvements were minor and class meaning declined. Therefore, the four-class model was selected as the most suitable representation of PGD symptom variation in both samples. Enumeration details are provided in Supporting Information (S1 Table and S2 Table).

### FMM results and model retention

Factor mixture models (FMM) combined the three-factor measurement structure with latent classes to test whether PGD symptoms reflected both dimensional and categorical features. In both samples, these models outperformed the CFA and LPA solutions, showing lower AIC and BIC values and improved class separation.

UK (N = 1,777): the three-factor, four-class model achieved the best balance of fit, parsimony, and interpretability (AIC = 42877.53, BIC = 43310.67, entropy = 1.00). Entropy values of 1.00 indicate deterministic class assignment in these data and should be interpreted as reflecting minimal overlap between severity-defined classes rather than evidence of discrete latent types. Although AIC and BIC declined slightly for the five- and six-class models (e.g., 3-factor 6-class AIC = 42082.60, BIC = 42658.29), gains were minimal and class clarity weakened. While the LMR-A test was statistically significant for some higher-class solutions (e.g., p = .025), these improvements were not accompanied by clearer class separation, stable symptom profiles, or improved interpretability. Item 7 (blame) showed unstable loadings across classes but retained clinical distinctiveness.

Republic of Ireland (N = 950): the three-factor, four-class FMM again provided the clearest and most stable structure (AIC = 27545.31, BIC = 27924.11, entropy = 1.00, p = .056). Five- and six-class models showed small numerical improvements (e.g., 3-factor 6-class AIC = 27335.15, BIC = 27840.22) but reduced interpretability, despite some statistically significant or borderline LMR-A results (e.g., p = .001). As in the UK sample, Item 7 (blame) showed cross-class instability but remained clinically meaningful in distinguishing high-severity groups.

**Fig 1:**
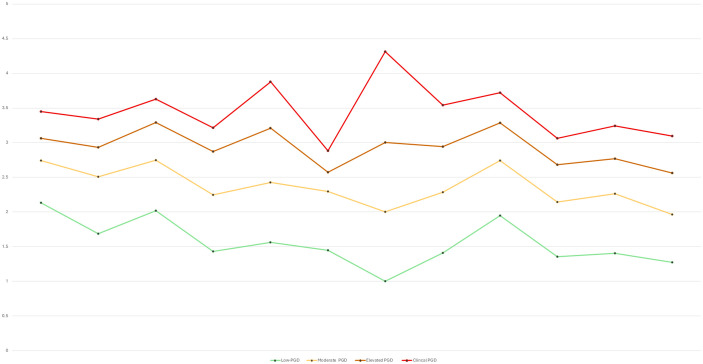
Profile plot of estimated intercepts for the four-class factor-mixture model solution (Sample 1: UK, N = 1,777).

**Fig 2:**
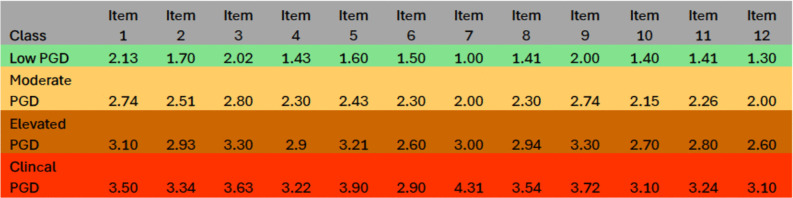
Estimated means of IPGDS items across the best-fitting factor-mixture model (Sample 1: UK, N = 1,777).

**Fig 3:**
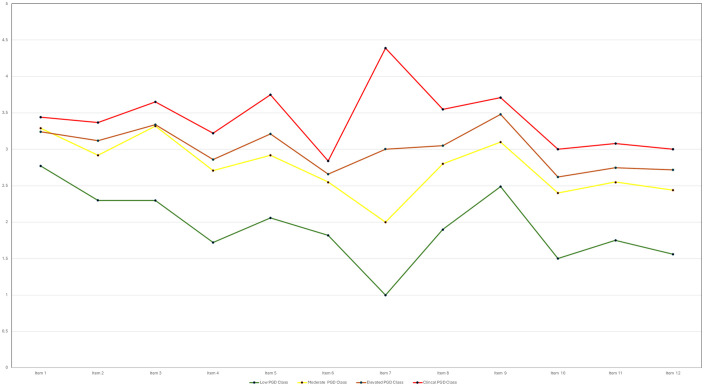
Profile plot of estimated intercepts for the four-class factor-mixture model solution (Sample 2: Ireland, N = 950).

**Fig 4:**
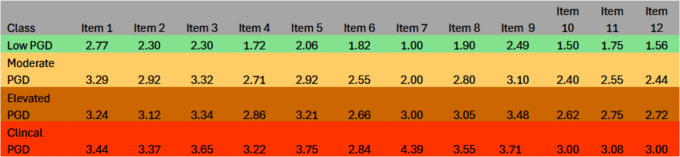
Estimated means of IPGDS items across the best-fitting factor-mixture model (Sample 2: Ireland, N = 950).

Overall, a three-factor, four-class FMM was retained in both samples for its strong fit, clear separation, and theoretical interpretability. Although the three-factor measurement structure could in principle be represented using a conventional structural equation diagram, such representations are less informative for factor mixture models, where latent dimensions and latent classes are estimated jointly and class-specific parameters are allowed. A single path diagram would therefore obscure the hybrid nature of the retained model by implying a purely dimensional structure. Instead, the organisation of symptoms is visualised using factor-informed profile plots and item-level class means (Figs 1–4), which directly display how the three dimensions and severity-ordered classes jointly shape symptom patterns. This approach provides a more transparent representation of the model than a simplified structural diagram would allow. Full selection statistics are presented in S1 Table and S2 Table.

### Class prevalence and classification accuracy

Class prevalence followed practical the same ordering in both samples.

UK: Low 68% [95% CI: 65–70%], Moderate 16% [14–18%], Elevated 10% [8–11%], Clinical 6% [5–7%]. Republic of Ireland: Low 69% [66–72%], Moderate 15% [13–17%], Elevated 11% [9–13%], Clinical 5% [4–6%].

Classification was highly precise. However, entropy can be inflated when latent classes represent ordered severity bands with little overlap in observed response distributions, and should not be taken as evidence of discrete latent types or as proof against model overfitting in a single sample. Entropy was 1.00, indicating near-perfect separation of classes and highly confident assignment of individuals to a single class. Class-specific average posterior probabilities were also very high in both samples (UK 0.984–0.992; Ireland ≥ 0.97), indicating negligible overlap. Although entropy values approaching 1.00 are uncommon in psychological applications, additional checks did not indicate artificial inflation. Posterior probabilities showed clear within-class assignment with minimal ambiguity, and repeated estimation with multiple random starts yielded identical solutions, supporting the robustness of class separation. Class prevalence and assignment metrics are summarised in Supporting Information (S1 Table and S2 Table). Additionally, time since loss was examined as an auxiliary distal outcome using the BCH method to assess whether bereavement timing differed across the severity-defined latent classes. In the UK sample, time since loss differed significantly across classes, whereas no significant differences were observed in the Republic of Ireland sample. Class-specific means and standard errors are reported in S4 Table.

### Symptom profiles and item-level patterns

For ease of interpretation, IPGDS items are discussed in three conceptual groupings: Items 1–4 (core separation distress), Items 5–8 (mid-band items: anger, avoidance, blame, acceptance), and Items 9–12 (remaining emotional and functional symptoms). Figs 1–4 display item-level means in their original order to preserve comparability across analyses.

Class means formed a clear severity gradient in both samples. In the UK sample, the Low class showed uniformly low endorsement across all symptoms. The Moderate class displayed modest increases, especially for separation-distress items (Items 1–3). The Elevated class showed substantial increases across longing, preoccupation, emotional pain, and difficulty moving on, while the Clinical class exhibited broad elevations with means above 3 for most items. The Irish sample reproduced the same pattern. Figs 1–4 display factor-informed profile plots and estimated item means by class. Across both samples, symptom profiles progressed in a largely monotonic fashion across classes, with each successive class showing incremental increases in symptom endorsement rather than qualitative reconfiguration of item patterns. No class displayed a distinct symptom configuration characterised by selective elevation or suppression of specific items independent of overall severity. Instead, differences between classes reflected graded intensity across the same symptom dimensions, supporting interpretation of the latent classes as severity bands rather than qualitatively distinct PGD subtypes.

All figs are plotted on the original IPGDS response scale (1–5), with identical y-axis scaling across samples to facilitate visual comparison of class profiles.

### Item level anomalies

Local measurement issues were concentrated in the mid-scale band (Items 5–8). Item 5 (anger) rose sharply with class severity but did not align cleanly with a single factor. Item 6 (avoidance) remained comparatively low and flat across classes, even in the Clinical group, and showed unstable loadings. Avoidance also appeared to behave differently across severity levels and sampling contexts. In community samples, behavioural avoidance may be constrained by ongoing social roles, environmental demands, or normative coping expectations, limiting its discriminative value even among individuals with elevated symptom severity. In contrast, avoidance has been shown to play a more central and impairing role in treatment-seeking or clinically defined grief presentations [6,16]. Together, these patterns suggest that certain IPGDS items may be more sensitive to severity thresholds or contextual factors than to latent structural distinctions per se. From a scale-development perspective, this highlights the importance of distinguishing core grief symptoms from severity-contingent or context-dependent indicators when refining PGD measurement for population surveillance versus clinical assessment purposes [28,41]. Item 7 (blame) was the strongest observed differentiator, and it was lowest in Low and highest in Clinical, but it showed weak or collapsing loadings across specifications. Item 8 (difficulty accepting) increased with severity but split across factors. These four items jointly produced the mid band instability that undermined strict factor models. From a clinical and phenomenological perspective, the observed instability across Items 5–8 aligns with longstanding debates regarding the role of cognitive–affective processes in prolonged grief. Persistent self- or other-blame may reflect high-severity maladaptive cognitions and affective dysregulation that emerge predominantly at the upper end of the grief continuum, rather than constituting a core symptom present across all severity levels [16,25]. The sharp differentiation of blame between the Elevated and Clinical classes is therefore consistent with evidence that such cognitions function as severity-linked amplifiers of distress rather than markers of qualitatively distinct subtypes of grief pathology [3,27]. The retained FMM accommodated this local misfit while preserving class clarity.

### Cross-cohort replication

The same three-factor structure, four latent classes, prevalence ordering, and mid-band instability replicated across the UK and Irish samples, including the distinctive behaviour of Items 5–8 and the strong elevation of blame in the Clinical class.

### Pairwise class comparisons

Planned comparisons between Clinical and Elevated classes showed higher scores for the Clinical group across nearly all symptoms in both samples. The largest differences were observed for anger, blame, difficulty accepting, and withdrawal from activities. Avoidance did not differ significantly between these two upper classes [23].

### Reliability and factor intercorrelations

Internal consistency was acceptable to excellent for all retained factors in both samples (α = .73–.86). Factor correlations were moderate to high, consistent with related dimensions within a shared construct [25]. Results were unchanged when the optimal CFA structure was re-estimated using WLSMV on polychoric correlations. Allowing class-specific factor variances in the FMM did not improve parsimony or stability and was not retained. Although variance heterogeneity can, in principle, capture meaningful subgroup distinctions, in the present data it produced no improvement in fit, interpretability, or replication, and did not reveal differential dispersion patterns suggestive of qualitatively distinct subtypes. Constraining variances therefore supported a more parsimonious model in which classes differed primarily in mean symptom severity rather than in structural heterogeneity.

## Discussion

Across two large independent population cohorts, our hybrid analyses (EFA, CFA, LPA, FMM) indicate that the IPGDS does not yet conform to a single confirmatory latent structure. EFAs showed cross-loadings and CFAs required post hoc modifications. LPA indices, including the monotonic decrease in BIC, did not provide a clear elbow. In practice, the most epidemiologically useful summary was a 3-factor, 4-class factor-mixture solution. This solution is best treated as descriptive rather than definitive, but it yielded stable class distributions with excellent assignment quality. The three correlated dimensions we recovered (separation distress, emotional reactivity, emotional numbing) are consistent with recent population work on ICD-11 PGD that has recovered comparable three-factor structures, supporting a primarily dimensional organization of symptoms at the population level [23,32]. This pattern is also broadly consistent with findings from international validation studies of the IPGDS and related ICD-11 PGD measures, which have reported similar multidimensional structures and severity-graded symptom distributions across culturally distinct population samples [26,28,35]. The replication of comparable factor and class solutions across UK and Irish cohorts therefore supports the cross-context generalisability of the core dimensional organisation of PGD symptoms, while allowing for local item-level variation. Factor-mixture modeling is well suited to this problem because it allows severity gradients and latent subgroups to coexist without overcommitting to either a purely dimensional or purely categorical account [12]. This pattern is consistent with taxometric and dimensional-spectrum accounts of psychopathology, which predict continuously distributed symptom severity with apparent subgroups emerging primarily as pragmatic severity bands rather than as qualitatively distinct syndromes.

Conceptually, the three dimensions we recovered correspond to well described clusters in contemporary grief theory. Separation distress reflects attachment related yearning for the deceased, persistent emotional pain, and preoccupation with the loss, which are central features of prolonged grief disorder as described in both diagnostic and theoretical models [1,2,4]. These symptoms are understood to reflect ongoing attachment needs and difficulty integrating the reality of the loss. Emotional reactivity captures heightened emotional responses such as anger, guilt, and difficulty regulating affect in response to loss related cues, consistent with cognitive and stress response models of grief related psychopathology [3,16,25]. Emotional numbness refers to a marked reduction or absence of emotional experience, feelings of emptiness and detachment, and a diminished sense of meaning, which are commonly described in severe or prolonged grief presentations [2,5]. Together, these dimensions closely align with symptom groupings identified across prior psychometric and clinical studies of prolonged grief disorder, supporting the substantive interpretability of the recovered factor structure.

At the dimensional level, both cohorts favored a three-factor solution over simpler alternatives however, with a reproducible local instability. Items 5–8 (anger, avoidance, blame and acceptance) showed weak, split, or diffuse loadings, with Item 7 (blame) demonstrating partial loading collapse across specifications. Outside this mid-band, item factor relations were coherent and replicated across cohorts. This pattern is compatible with a largely quantitative severity scaffold on which a small set of indicators behave atypically at the upper tail of severity, an observation in line with recent structural work on ICD-11 PGD [23,32]. Notably, similar forms of item-level instability have been reported in prior psychometric studies of prolonged grief and related trauma-related constructs, particularly for cognitively complex or behaviourally contingent indicators such as blame, avoidance, and acceptance difficulties [23,26,29]. This convergence suggests that mid-band instability may reflect a broader psychometric challenge inherent to measuring higher-order cognitive–affective processes in grief, rather than a sample-specific artefact. At the same time, cultural norms around responsibility, coping, and emotional expression may influence how these items perform across populations, underscoring the importance of continued cross-cultural evaluation.

At the class level, four classes (low, moderate, elevated, clinical) were recovered in each cohort and formed a clear severity gradient; most symptoms rose in parallel from Low to clinical. Model selection tests (LMR-A and information criteria) did not point to a single best number of classes, as model fit kept improving slightly with more classes. Combined with the strong classification accuracy (entropy), this suggests the classes represent practical severity levels rather than truly distinct subtypes, consistent with previous grief studies showing that groups mainly differ by symptom intensity rather than by qualitatively different profiles [7,21,30]. In both cohorts, delineation of the clinical class was disproportionately driven by a narrow symptom band, especially blame (Item 7), implying that thresholding on a few high-severity indicators can produce apparent class boundaries even when a single severity continuum predominates.

Item-level profiles sharpen this picture. In both cohorts, Items 1–4 and 8–12 increased roughly in parallel across classes, consistent with a mostly quantitative shift. In contrast, the mid-band accounted for the measurement failure in strict factor models. Anger (Item 5) surged only in the top class, suggesting a nonlinear jump at extreme severity. Avoidance (Item 6) stayed relatively low and did not distinguish well between classes, even at the clinical level. Acceptance (Item 8) increased with severity but failed to align with a single factor, while blame (Item 7) was minimal in the low and moderate classes yet reached its highest levels in the clinical class, contributing little to the factor structure.

Psychometrically, blame (Item 7) behaves less like a stable indicator of a latent reactivity dimension and more like a high-severity flag. Clinically, endorsement of persistent blame should trigger concern for a severe PGD presentation. Similar patterns are seen in trauma-related conditions, where the most severe subgroups display externalised anger and moral injury features, and at the highest levels, reduced avoidance or emotional flooding [17,18]. These findings have both epidemiological and clinical implications. From a translational perspective, the four severity-based classes provide a pragmatic framework for stepped care and triage rather than categorical diagnosis. Individuals in the Low and Moderate classes are likely to benefit from monitoring, psychoeducation, or low-intensity interventions, whereas those in the Elevated class may warrant targeted preventive support. The Clinical class, characterised by high overall severity and disproportionate endorsement of high-risk indicators such as blame and anger, represents a group for whom specialist assessment and intensive intervention are most clearly indicated. In this way, class membership can inform proportional allocation of resources while preserving the underlying dimensional nature of PGD symptoms. For epidemiological surveillance, the present findings suggest value in combining continuous severity scores with a small number of high-risk indicators when identifying individuals most in need of follow-up. While total symptom severity captures the graded distribution of PGD in the population, items such as persistent blame or intense anger appear to function as severity-linked risk markers that disproportionately characterise the highest-need group. Surveillance systems that integrate dimensional scoring with such flags may therefore improve sensitivity to clinically significant distress without imposing rigid diagnostic thresholds, supporting more responsive public-health monitoring. For surveillance and triage, screening tools that combine core separation-distress items with direct questions on persistent blame and anger may better identify the highest-risk individuals, while avoidance adds little to distinguishing the most severe cases in general-population samples. For treatment planning, the provisional dimensions align with plausible intervention targets: acceptance and meaning-focused approaches for separation distress, behavioural activation for numbing and disengagement, and cognitive or acceptance-based strategies addressing hostile and self-blaming thoughts (including, where appropriate, forgiveness-focused elements) for anger and blame presentations [2,13,30].

At the instrument level, cross-cohort convergence supports the IPGDS as a useful population measure of PGD domains, however, the repeated instability of Items 5–8 and the category/dimension mismatch for blame require attention. Practically, researchers and clinicians should treat blame (and possibly anger) as risk signposts rather than as stable anchors for longitudinal scaling. Conceptually, focused refinement of the mid-band items is needed, including careful review of item wording, response scaling, and conceptual clarity to ensure they capture distinct aspects of grief rather than overlapping content. Future work should also include explicit tests of partial invariance across severity levels and assess whether blame functions better as a diagnostic specifier or auxiliary severity marker, rather than as a core factor indicator, within mixture modeling frameworks [12]. Additionally, time since bereavement was examined post hoc as a distal outcome using the BCH approach. Results indicated that time since loss differed across severity-defined classes in the UK sample but not in the Republic of Ireland sample (S4 Table). These mixed findings suggest that while bereavement timing may contribute to class separation in some population contexts, it does not appear to be a robust or consistent driver of severity-defined class membership across cohorts.

Several limitations temper inference. First, both samples were drawn from non-clinical, self-selected online panels, which imposes important constraints on generalisability. Although this design is appropriate for population-level epidemiological analysis, it is likely to underrepresent individuals with the most severe or complex PGD presentations, particularly those who are disengaged from online research, lack stable internet access, or are currently receiving intensive clinical care. As a result, the prevalence and composition of the highest-severity class may differ in treatment-seeking or service-connected populations. Second, online recruitment may differentially exclude marginalised bereaved individuals, including those facing socioeconomic disadvantage, lower digital literacy, language barriers, or intersecting stressors related to migration or minority status. These factors may influence both symptom expression and item functioning, particularly for cognitively or culturally nuanced indicators such as blame, avoidance, and acceptance. Accordingly, the present findings should be interpreted as describing the latent organisation of PGD symptoms in community-dwelling, digitally accessible adults rather than as definitive representations of clinical populations. Finally, the cross-sectional design cannot determine whether the Elevated and Clinical classes reflect stable trajectories or transient states. Small Clinical classes also inflate uncertainty. Parsimony choices, such as constraining factor variances equal across classes, may mask subtle non-invariance. The collective signs of instability across EFA, CFA, LPA, and FMM argue against strong structural claims at this stage.

Future work should prioritize longitudinal designs to test class stability and transitions and to evaluate the persistence of anger and blame as prognostic markers [43,44]. Focused item-level refinement and DIF testing for Items 5–8 (with partial invariance modeling where justified) should be paired with mixture-based comparisons of ICD-11 versus DSM-5-TR duration thresholds and with external validation using functional impairment and service-use outcomes. Replication in treatment-seeking samples can test whether the severe class is overrepresented and whether mid-band behavior changes with case mix. Trials that stratify by baseline profiles or dominant dimensions can evaluate whether tailoring treatment to profile improves outcomes. Reporting standards should frame FMMs as descriptive maps of symptom organization, include sensitivity checks for class counts, and preregister hypotheses about the mid-band.

In conclusion, evidence from two national cohorts supports a 3-factor, 4-class factor mixture as the most informative descriptive account of PGD symptoms. Most indicators vary continuously with severity, while blame and anger act as high-severity markers and avoidance contributes little at peak severity. These patterns favor a primarily dimensional structure with classes used as practical severity groupings, and on that basis the hybrid, severity anchored account is immediately useful for epidemiology, screening, and stepped care [7,21,23,30,32]. Definitive structural claims remain premature until Items 5–8 are refined and partial invariance is demonstrated; priorities include longitudinal tests of class stability and transitions, DIF and partial invariance checks for Items 5–8, external validation on impairment and service use, and replication in treatment-seeking samples.

## Supporting information

S1 Table Estimated class-profile plots from the retained three-factor, four-class factor-mixture model (Sample 1: UK, N = 1,777). (DOCX)

S2 Table Estimated class-profile plots from the retained three-factor, four-class factor-mixture model (Sample 2: ROI, N = 950). (DOCX)

S3 Table Standardized CFA factor loadings (STDYX) for the retained three-factor IPGDS model in Irish and UK samples. (DOCX)

S4 Table Time since loss across latent classes in the three-factor, four-class model in UK and Republic of Ireland samples. (DOCX)

S1 STROBE Checklist Completed STROBE checklist for cross-sectional observational studies. (DOCX)
