# Supplementary material for: The latent structure of ICD-11 Prolonged Grief: Replicated factor mixture models in two national cohorts
Source: PLOS Ment Health. 2026 Feb 20;3(2):e0000515. doi: 10.1371/journal.pmen.0000515 (PMC12923040; doi:10.1371/journal.pmen.0000515)
Supplement: S1 Table — (DOCX) [file pmen.0000515.s001.docx]

S1 Table presents the fit indices for the EFA, CFA, LPA, and FMM approaches applied to the International Prolonged Grief Disorder Scale (IPGDS) in Sample 1: UK C19PRC-UK

| Model | Log-likelihood | χ^2^ (df)  p | CFI  TLI | RMSEA | SRMR | AIC | BIC | Entropy | LMR-A (p) |
| --- | --- | --- | --- | --- | --- | --- | --- | --- | --- |
| EFA |  |  |  |  |  |  |  |  |  |
| 1 Factor | -24800.381 | 1326.824 (54) p < .001 | .854 .822 | .116 | .056 | 49672.761 | 49870.138 |  |  |
| 2 Factor | -23995.807 | 406.833 (43)  p < .001 | .958 .936 | .069 | .029 | 48085.615 | 48343.301 |  |  |
| 3 Factor | -23759.288 | 128.770 (33) p < .001 | .989 .978 | .040 | .012 | 47632.575 | 47945.088 |  |  |
| 4 Factor | -23687.208 | 40.443 (24) p =.002 | .998  .995 | .020 | .007 | 47506.416 | 47868.273 |  |  |
|  |  |  |  |  |  |  |  |  |  |
| CFA |  |  |  |  |  |  |  |  |  |
| Model 2 (one-factor) | -24800.381 | 1302.283 (53) p < .001 | .857 .822 | .115 | .056 | 49674.761 | 49877.620 |  |  |
| Model 4 (two-factor) | -24312.742 | 751.662 (51)  p < .001 | .920 .896 | .088 | .054 | 48703.484 | 48917.309 |  |  |
| Model 5 (two-factor cross-loading item 4) | -25037.658 | 1622.962 (53)  p < .001 | .820 .776 | .129 | .224 | 50149.315 | 50352.175 |  |  |
| Model 6 (three-factor) cross-loading(item 8 Spain) | -24117.800 | 558.328(53) p < .001 | .942  .928 | .073 | .092 | 48309.599 | 48512.458 |  |  |
| Model 7 (three-factor) cross-loading (item 8) (ESEM) | -24069.409 | 499.777 (53) p < .001 | .949 .936 | .069 | .105 | 48212.818 | 48415.678 |  |  |
| Model 7 (three-factor) Factor based on EFA | 24106.90 | 540.236 (53) p < .001 | .944  .932 | .071 | .164 | 48285.80 | 48483.17 |  |  |
| Model 9 (4 factor: Fragmentation) | -23,993.479 | 403.188 (48) p < .001 | .959 .944 | .065 | .036 | 48070.958 | 48301.231 |  |  |
| LPA |  |  |  |  |  |  |  |  |  |
| 2 classes | -26273.165 | N/A | N/A | N/A | N/A | 52620.330 | 52823.189 | 0.963 | 10664.785 p < .001 |
| 3 classes | -24759.717 | N/A | N/A | N/A | N/A | 49619.434 | 49893.568 | 0.933 | 2996.095 p < .001 |
| 4 classes | -24032.927 | N/A | N/A | N/A | N/A | 48191.853 | 48537.262 | 0.930 | 1438.790 p < .001 |
| 5 classes | -23658.029 | N/A | N/A | N/A | N/A | 47468.058 | 47884.742 | 0.929 | 742.166 p= 0.1565 |
| 6 classes | -22855.185 | N/A | N/A | N/A | N/A | 45888.371 | 46376.330 | 0.963 | 641.649 p < .001 |
|  |  |  |  |  |  |  |  |  |  |
| FMM |  |  |  |  |  |  |  |  |  |
| 1 factor 2 classes | -23782.706 | N/A | N/A | N/A | N/A | 47663.412 | 47932.063 | .943 | 2014.638 p < .001 |
| 2 factor 2 classes | -23343.473 | N/A | N/A | N/A | N/A | 46786.946 | 47061.081 | .995 | 1918.812 p < .001 |
| 3 factor 2 classes | -23122.988 | N/A | N/A | N/A | N/A | 46351.975 | 46642.558 | .997 | 1790.307 p < .001 |
| 1 factor 3 classes | -23291.437 | N/A | N/A | N/A | N/A | 46706.874 | 47046.800 | .943 | 972.540 p=.001 |
| 2 factors 3 classes | -22897.239 | N/A | N/A | N/A | N/A | 45920.478 | 46265.887 | .997 | 883.387  p < .001 |
| 3 factors 3 classes | -22676.326 | N/A | N/A | N/A | N/A | 45484.652 | 45846.509 | .997 | 884.234  p < .001 |
| 1 factor 4 classes | -22804.327 | N/A | N/A | N/A | N/A | 45758.654 | 46169.856 | .967 | 964.306 p < .001 |
| 2 factors 4 classes | -21574.730 | N/A | N/A | N/A | N/A | 43301.461 | 43718.145 | 1.000 | 2618.103 p=.111 |
| 3 factors 4 classes | -21359.767 | N/A | N/A | N/A | N/A | 42877.533 | 43310.665 | 1.000 | 2606.325 p=.110 |
| 1 factor 5 classes | -21594.506 | N/A | N/A | N/A | N/A | 43365.012 | 43847.488 | .926 | 907.637 p < .001 |
| 2 factors 5 classes | -21252.921 | N/A | N/A | N/A | N/A | 42683.841 | 43171.800 | .974 | 637.070 p < .001 |
| 3 factors 5 classes | -21105.716 | N/A | N/A | N/A | N/A | 42395.431 | 42899.838 | .977 | 502.932 p=.001 |
| 1 factor 6 classes | -21406.391 | N/A | N/A | N/A | N/A | 43014.781 | 43568.532 | .915 | 372.402 p= .25 |
| 2 factors 6 classes | -21091.861 | N/A | N/A | N/A | N/A | 42387.722 | 42946.956 | .969 | 318.841 p=.22 |
| 3 factors 6 classes | -20936.302 | N/A | N/A | N/A | N/A | 42082.604 | 42658.285 | .972 | 335.380 p=.025 |

Note: AIC =Akaike Information Criterion, BIC =Bayesian Information Criterion, LMR-A Lo-Mendell- Rubin adjusted likelihood ratio test. Best-fitting models for each approach (EFA, CFA, LPA, FMM) shown in bold
