# Supplementary material for: The latent structure of ICD-11 Prolonged Grief: Replicated factor mixture models in two national cohorts
Source: PLOS Ment Health. 2026 Feb 20;3(2):e0000515. doi: 10.1371/journal.pmen.0000515 (PMC12923040; doi:10.1371/journal.pmen.0000515)
Supplement: S2 Table — (DOCX) [file pmen.0000515.s002.docx]

S2 Table presents the fit indices for the EFA, CFA, LPA, and FMM approaches applied to the International Prolonged Grief Disorder Scale (IPGDS) in Sample 2: Republic of Ireland (N = 950)

| Model | Log-likelihood | χ^2^ (df)  p | CFI  TLI | RMSEA | SRMR | AIC | BIC | Entropy | LMR-A (p) |
| --- | --- | --- | --- | --- | --- | --- | --- | --- | --- |
| EFA |  |  |  |  |  |  |  |  |  |
| 1 Factor | -15239.570 | 891.672 (54) p < .001 | .840 .084 | .128 | .062 | 30551.14 | 30725.97 |  |  |
| 2 Factor | -14802.469 | 231.082 (43)  p < .001 | .964  .945 | .068 | 0.30 | 29698.94 | 29927.19 |  |  |
| 3 Factor | -14706.274 | 80.175 (33) p < .001 | .991 .982 | .039 | .013 | 29526.55 | 29803.37 |  |  |
| 4 Factor | -14684.939 | 48.766 (24) p =.002 | .995 .987 | .033 | .010 | 29501.88 | 29822.40 |  |  |
|  |  |  |  |  |  |  |  |  |  |
| CFA |  |  |  |  |  |  |  |  |  |
| Model 2 (one-factor) | -15239.570 | 891.668 (54) p < .001 | .840 .804 | .128 | .062 | 30551.14 | 30725.97 |  |  |
| Model 3 (two-factor) | -15010.763 | 556.149(55)  p < .001 | .904 .885 | .098 | .125 | 30091.526 | 30261.502 |  |  |
| Model 4 Three Factor based on EFA | −14876.630 | 349.351 (54) p < .001 | .944 .931 | .076 | .122 | 29825.26 | 30000.09 |  |  |
| Model 5 Three Factor based on EFA – item 8 taken out | -15300.436 | 1016.662(55) p < .001 | .816  .779 | .136 | .229 | 30670.871 | 30840.847 |  |  |
| LPA |  |  |  |  |  |  |  |  |  |
| 2 classes | -15988.875 | N/A | N/A | N/A | N/A | 32051.75 | 32231.44 | .943 | 4495.25 p < .001 |
| 3 classes | -15369.530 | N/A | N/A | N/A | N/A | 30839.06 | 31081.88 | .906 | 1224.948 p= .063 |
| 4 classes | ‑14957.558 | N/A | N/A | N/A | N/A | 30 041.12 | 30 347.07 | .900 | 814.80 p= .017 |
| 5 classes | -14771.228 | N/A | N/A | N/A | N/A | 29694.46 | 30063.55 | .894 | 368.525 p= 0.051 |
| 6 classes | -14650.850 | N/A | N/A | N/A | N/A | 29479.70 | 29911.93 | .878 | 238.09 p= .293 |
|  |  |  |  |  |  |  |  |  |  |
| FMM |  |  |  |  |  |  |  |  |  |
| 1 factor 2 classes | -14751.639 | N/A | N/A | N/A | N/A | 29601.278 | 29839.245 | .913 | 965.036 p < .001 |
| 2 factor 2 classes | -14837.236 | N/A | N/A | N/A | N/A | 29778.472 | 30031.008 | .862 | 5131.717 p < .001 |
| 3 factor 2 classes | -15653.645 | N/A | N/A | N/A | N/A | 31411.290 | 31663.827 | .931 | 2672.927 p < .001 |
| 1 factor 3 classes | -14565.061 | N/A | N/A | N/A | N/A | 29254.122 | 29555.223 | .929 | 369.016 p=.026 |
| 2 factors 3 classes | -14454.414 | N/A | N/A | N/A | N/A | 29042.829 | 29368.212 | .903 | 758.270 p = .188 |
| 3 factors 3 classes | -15233.007 | N/A | N/A | N/A | N/A | 30602.014 | 30932.253 | .867 | 833.677 p= .016 |
| 1 factor 4 classes | -14117.275 | N/A | N/A | N/A | N/A | 28384.550 | 28748.785 | 1.00 | 885.636 p = .004 |
| 2 factors 4 classes | -14454.414 | N/A | N/A | N/A | N/A | 29072.829 | 29471.059 | .923 | P= 0.24 |
| 3 factors 4 classes | -13694.652 | N/A | N/A | N/A | N/A | 27545.305 | 27924.109 | 1.00 | 1062.778  p= .056 |
| 1 factor 5 classes | -13865.418 | N/A | N/A | N/A | N/A | 27906.835 | 28334.204 | .915 | 432.321 p < .001 |
| 2 factors 5 classes | -14337.080 | N/A | N/A | N/A | N/A | 27979.813 | 28450.890 | .999 | 1058.827 p = .056 |
| 3 factors 5 classes | -13615.071 | N/A | N/A | N/A | N/A | 27412.142 | 27854.080 | .943 | 157.397 p=.001 |
| 1 factor 6 classes | -13763.881 | N/A | N/A | N/A | N/A | 27729.762 | 28220.264 | .917 | 200.821 p= .76 |
| 2 factors 6 classes | -14578.951 | N/A | N/A | N/A | N/A | 28009.813 | 28553.736 | .999 | 1146.770  p= .24 |
| 3 factors 6 classes | -13563.573 | N/A | N/A | N/A | N/A | 27335.147 | 27840.219 | .937 | 101.853 p=.82 |
