# Supplementary material for: The latent structure of ICD-11 Prolonged Grief: Replicated factor mixture models in two national cohorts
Source: PLOS Ment Health. 2026 Feb 20;3(2):e0000515. doi: 10.1371/journal.pmen.0000515 (PMC12923040; doi:10.1371/journal.pmen.0000515)
Supplement: S3 Table — (DOCX) [file pmen.0000515.s003.docx]

S3 Table. Standardized CFA factor loadings (STDYX) for the retained three factor IPGDS model in Irish and UK samples

| IPGDS Item | Factor | Irish (N = 950) | UK (N = 1777) |
| --- | --- | --- | --- |
| 1. I am longing or yearning for the deceased. | Separation distress | 0.829 | 0.837 |
| 1. I am preoccupied with thoughts about the deceased or circumstances of the death. | Separation distress | 0.873 | 0.875 |
| 1. I have intense feelings of sorrow, related to the deceased. | Separation distress | 0.871 | 0.915 |
| 1. I feel guilty about the death or circumstances surrounding the death. | \| Emotional reactivity \| \| --- \|  \|  \| \| --- \| | 0.736 | 0.816 |
| 1. I am angry over the loss. | Emotional reactivity | 0.804 | 0.860 |
| 1. I try to avoid reminders of the deceased or the death as much as possible (e.g., pictures, memories). | Emotional numbness | 0.684 | 0.754 |
| 1. I blame others or the circumstances for the death (e.g., a higher power). | Emotional reactivity | 0.579 | 0.734 |
| 1. I have trouble or just do not want to accept the loss. | Emotional reactivity | 0.854 | 0.889 |
| 1. I feel that I lost a part of myself. | Separation distress | 0.799 | 0.829 |
| 1. I have trouble or have no desire to experience joy or satisfaction. | Emotional numbness | 0.887 | 0.900 |
| 1. I feel emotionally numb. | Emotional numbness | 0.899 | 0.926 |
| 1. I have difficulties engaging in activities I enjoyed prior to the death. | Emotional numbness | 0.901 | 0.914 |

Note. Values are standardized factor loadings (STDYX) from MLR CFA models. Factor labels match the manuscript: Separation distress, Emotional reactivity, Emotional numbness.
