# Supplementary material for: The latent structure of ICD-11 Prolonged Grief: Replicated factor mixture models in two national cohorts
Source: PLOS Ment Health. 2026 Feb 20;3(2):e0000515. doi: 10.1371/journal.pmen.0000515 (PMC12923040; doi:10.1371/journal.pmen.0000515)
Supplement: S4 Table — (DOCX) [file pmen.0000515.s004.docx]

S4 Table. Time Since Loss Across Latent Classes in the 3-Factor, 4-Class Model in UK and Republic of Ireland Samples

UK Sample (N = 1,777)

| Latent Class | Approx. N | Mean | SE |
| --- | --- | --- | --- |
| Low | 1,208 | 4.337 | 0.171 |
| Moderate | 284 | 4.925 | 0.041 |
| Elevated | 178 | 4.493 | 0.088 |
| Clinical | 107 | 4.558 | 0.143 |
| Omnibus test: χ²(3) = 31.001, p < .001 |  |  |  |
| Republic of Ireland Sample (N = 950) |  |  |  |
| Low | 655 | 5.663 | 0.143 |
| Moderate | 143 | 5.130 | 0.185 |
| Elevated | 105 | 5.468 | 0.061 |
| Clinical | 47 | 5.321 | 0.146 |
| Omnibus test: χ²(3) = 6.098, p = .107 |  |  |  |

Note. Time since loss was examined across latent classes using the BCH distal outcome method in Mplus. Means and standard errors are BCH-adjusted. Class sizes are approximate and derived from model-estimated class prevalences and total sample size. The UK sample showed significant differences in time since loss across classes, whereas no significant differences were observed in the Republic of Ireland sample.
