# Supplementary material for: The latent structure of ICD-11 Prolonged Grief: Replicated factor mixture models in two national cohorts
Source: PLOS Ment Health. 2026 Feb 20;3(2):e0000515. doi: 10.1371/journal.pmen.0000515 (PMC12923040; doi:10.1371/journal.pmen.0000515)
Supplement: S1 STROBE Checklist — (DOCX) [file pmen.0000515.s005.docx]

**S1 STROBE Checklist. STROBE Statement checklist of items that should be included in reports of observational studies**

1a. Title/Abstract design term → Title page; Abstract (“two-cohort, cross-sectional analyses; factor-mixture modeling”).

1b. Informative abstract = Abstract.

2. Background/rationale = Introduction (paras 1–4).

3. Objectives = Introduction (final paragraph).

4. Study design (key elements) = Methods 2.4 Statistical Analysis.

5. Setting/dates = Methods 2.1 Participants (UK: Mar–Apr 2021; IE: Apr–Sep 2022; recruitment routes).

6. Cross-sectional eligibility/selection = Methods 2.1 (≥6 months since bereavement; panel sources; exclusions).

7. Variables/diagnostic criteria = Methods 2.3 Measures (IPGDS; symptom items modeled).

8. Data sources/measurement = Methods 2.3; rescaling in Irish sample.

9. Bias (how addressed) = Methods 2.4 (FIML, list-wise screening, replication across cohorts).

10. Study size (how arrived at) = Methods 2.1 (fixed panels; all eligible retained).

11. Quantitative variables handling = Methods 2.3 (0–4 to 1–5 rescale); 2.4 (no transformations needed).

12. Statistical methods = Methods 2.4 (EFA, CFA, LPA, FMM; selection criteria).

12b. Subgroups/interactions = Results 3.8 (Clinical vs Elevated comparisons).

12c. Missing data = Methods 2.4 (FIML; covariance coverage 1.00).

12e. Sensitivity analyses = Methods 2.4 (WLSMV re-estimation; variance constraints check).

13. Participants flow = Methods 2.1; Results 3.5 (class prevalence).

14. Descriptive data = Methods 2.2; Tables 1–2 (sample characteristics).

15. Outcome data = Results 3.1–3.6; Figures 1–4; Tables 3–5 (symptoms/classes).

16. Main results (estimates/precision) = Results 3.4–3.6 (class sizes, profiles; planned reporting sans global fit indices).

17. Other analyses = Results 3.7 replication; 3.9 robustness.

18. Key results vs aims = Discussion (opening).

19. Limitations = Discussion (limitations paragraph).

20. Interpretation = Discussion (measurement-first, cautious).

21. Generalisability = Discussion (population surveillance framing).

22. Funding = Funding statement (Ending section).

Ethics & consent = Methods 2.1 (ethics approvals; consent).

Data availability = Data Availability statement.
